# The Use of Graphene Oxide in Orthodontics—A Systematic Review

**DOI:** 10.3390/jfb14100500

**Published:** 2023-10-09

**Authors:** Joanna Rygas, Jacek Matys, Magdalena Wawrzyńska, Maria Szymonowicz, Maciej Dobrzyński

**Affiliations:** 1 Dental Practice, Wojciecha z Brudzewa 10, 51-601 Wroclaw, Poland; joanna.rygas@gmail.com; 2 Oral Surgery Department, Wroclaw Medical University, Krakowska 26, 50-425 Wroclaw, Poland; 3 Department of Orthodontics, Technische Universitat Dresden, 01307 Dresden, Germany; 4 Pre-Clinical Research Centre, Wroclaw Medical University, Bujwida 44, 50-345 Wroclaw, Poland; magdalena.wawrzynska@umw.edu.pl (M.W.); maria.szymonowicz@umw.edu.pl (M.S.); 5 Department of Pediatric Dentistry and Preclinical Dentistry, Wroclaw Medical University, Krakowska 26, 50-425 Wroclaw, Poland; maciej.dobrzynski@umw.edu.pl

**Keywords:** adhesive remnant index, antibacterial effect, corrosion resistance, demineralization, shear bond strength, white spots

## Abstract

Background: Graphene-based materials have great prospects for application in dentistry and medicine due to their unique properties and biocompatibility with tissues. The literature on the use of graphene oxide in orthodontic treatment was reviewed. Methods: This systematic review followed the PRISMA protocol and was conducted by searching the following databases: PubMed, Scopus, Web of Science, and Cochrane. The following search criteria were used to review the data on the topic under study: (Graphene oxide) AND (orthodontic) ALL FIELDS. For the Scopus database, results were narrowed to titles, authors, and keywords. A basic search structure was adopted for each database. Initially, a total of 74 articles were found in the considered databases. Twelve articles met the inclusion criteria and were included in the review. Results: Nine studies demonstrated the antibacterial properties of graphene oxide, which can reduce the demineralization of enamel during orthodontic treatment. Seven studies showed that it is biocompatible with oral tissues. Three studies presented that graphene oxide can reduce friction in the arch-bracket system. Two studies showed that it can improve the mechanical properties of orthodontic adhesives by reducing ARI (Adhesive Remnant Index). Three studies demonstrated that the use of graphene oxide in the appropriate concentration can also increase the SBS (shear bond strength) parameter. One research study showed that it can increase corrosion resistance. One research study suggested that it can be used to accelerate orthodontic tooth movement. Conclusion: The studies included in the systematic review showed that graphene oxide has numerous applications in orthodontic treatment due to its properties.

## 1. Introduction

**Figure 1:**
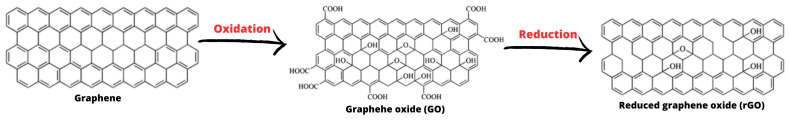
The figure illustrates the structural composition of graphene, graphene oxide (GO), and reduced graphene oxide (rGO) (created with Canva.com (accessed on 1 September 2023)).

Discovered in 2004, graphene oxide (GO) is considered one of the most promising nanomaterials. This two-dimensional carbon material acts as the fundamental graphite unit and is composed of a single layer of hexagonally arranged sp2 carbon atoms [1,2]. Furthermore, graphene possesses extraordinary characteristics, including a significant surface area and exceptional mechanical, electrical, and thermal properties [3,4,5]. These attributes enable graphene to be utilized in diverse applications [1,6]. Nevertheless, the application of graphene may encounter restrictions due to issues such as agglomeration and challenges in processing. To overcome these limitations, chemical modification becomes necessary in order to generate derivatives of graphene, like graphene oxide (GO) and reduced graphene oxide (rGO). These modified forms prove to be more versatile and find applications across various fields [1,6,7,8,9,10,11]. The provided illustration depicts the structures of graphene, graphene oxide (GO), and reduced graphene oxide (rGO). GO is derived from the oxidation process applied to graphene, whereas rGO is obtained through the chemical or thermal reduction of GO [1] (Figure 1).

**Figure 2:**
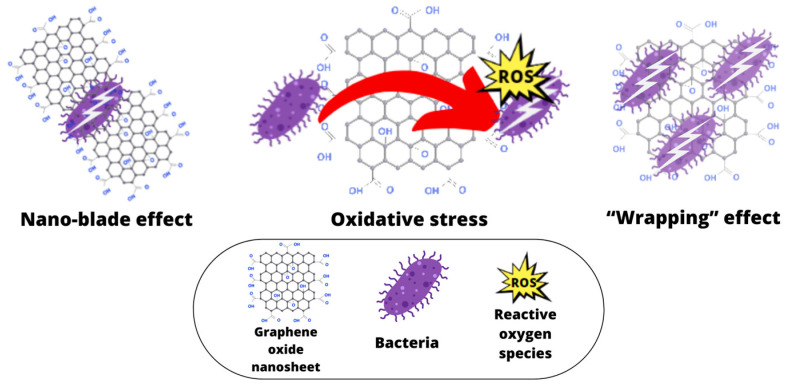
The diagram illustrates three distinct antibacterial mechanisms of graphene oxide (created with Canva.com (accessed on 1 September 2023)).

The majority of research conducted on the antimicrobial properties of graphene-based nanomaterials (GBNs) has proposed three potential mechanisms to explain their antimicrobial activity against various microorganisms [1,12,13]. The first is the induction of oxidative stress in bacterial cells through the formation of reactive oxygen species, which damage bacterial cell membranes and thereby disrupt their metabolic activity [1,7,14,15,16]. The second involves killing pathogens with the sharp edges of graphene oxide particles—the so-called “nano-sharpening effect” [1,3,14]. It causes leakage of the bacterial cytoplasmic fluid and consequently, cell death. The third is the isolation of the bacterial cell from the external environment—the so-called “wrapping effect” [1,7,14]. Graphene oxide nanoparticles wrap around bacterial cells and isolate them from the environment, thus preventing bacterial proliferation and cell membrane activity [1,14]. The mechanical disruption caused by electrostatic forces can result in the disturbance of bacterial cells, leading to alterations in membrane potential, depolarization, and compromised integrity of the bacterial cell membrane. This process eventually leads to osmotic imbalance, disruption of cellular respiration, cell lysis, and ultimately, the demise of the bacterial cell [1] (Figure 2).

The bactericidal effect of graphene oxide is influenced not only by intrinsic and extrinsic factors but also by the composition, structure, and maturity stage of microbial cells [1,7]. Researchers have acknowledged the impact of bacterial structure on antimicrobial activity. Understanding the underlying mechanisms of graphene-based nanomaterials can contribute to the development of dental materials resistant to microbial infections [1,8]. Nano-scale nanoparticles possess a higher surface area to volume ratio compared to non-nano particles, allowing for closer interaction with microbial membranes and enhanced antimicrobial activity [17,18]. To enhance antimicrobial properties, dental materials often incorporate antimicrobial agents such as chlorhexidine and quaternary ammonium compounds [1]. However, these additions often result in a compromise between antimicrobial effectiveness and mechanical properties [1,7]. In contrast, graphene oxide has the unique ability to associate with other biomaterials like polymers, ceramics, and metals, facilitating the design of biomaterials with desired properties. For instance, graphene and its derivatives can be incorporated into dental materials through methods such as colloidal dispersion, direct synthesis, sintering, and conjugation [1]. 

Recently, the use of graphene-oxide-based materials has become very beneficial in the field of dental research [7,8,19]. This is due to its favourable biological properties, high specific surface area, biocompatibility, physico-chemical stability, mechanical strength, electronic properties, and easy synthesis process, while also being low-cost [1,7,20]. Moreover, thanks to the mechanisms of antibacterial effect, graphene oxide can reduce the total amount of *S. mutans* (*Streptococcus mutans*) in the oral cavity and reduce the formation of white spot lesions [4,14,16,21]. This not only reduces the risk of carious cavities, but also improves the aesthetic effect at the end of orthodontic treatment [14,16]. It is also essential for orthodontists to assess the impact of graphene on the bonding of brackets to the tooth surface. Due to these properties, graphene oxide may not only have a wide range of uses in dentistry, but also in medicine. In recent studies [14,16], GO has been identified as a potential carrier in nanomedicine, especially for cancer treatment and controlled drug delivery systems. It can be an alternative to fight against bacterial infection [22,23]. Furthermore, recent research in the field of dental materials suggests the potential of graphene oxide usage as a sealer component in endodontic treatment [24]. The failure of endodontic treatment is directly associated with microbial infection in the root canal or periapical areas. An endodontic sealer that is both bactericidal and biocompatible is essential for the success of a root canal. The study [16] showed that graphene-oxide-based composites show promise as endodontic sealers for protection against reinfection in root canal treatment and enhance success in endodontic treatment overall treatments.

The main objective of this systematic review was to explore the impact of graphene oxide properties on its use in orthodontic treatment. Based on the analysed articles on the use of graphene oxide in orthodontic treatment, it was concluded that, due to its unique properties and biocompatibility, it is worth writing a systematic review on this topic. Furthermore, a systematic review on this topic has not yet been published. Such a review of the literature may encourage researchers to carry out further studies, which could be of great benefit to both orthodontists and patients in the future.

## 2. Materials and Methods

### 2.1. Focused Question

The systematic review followed the PICO framework as follows.

PICO question: in the case of orthodontic materials (population), will the addition of graphene oxide (investigated condition) cause a change in their properties (outcome) compared to orthodontic materials without the addition of graphene (comparison condition)?

### 2.2. Protocol

**Figure 3:**
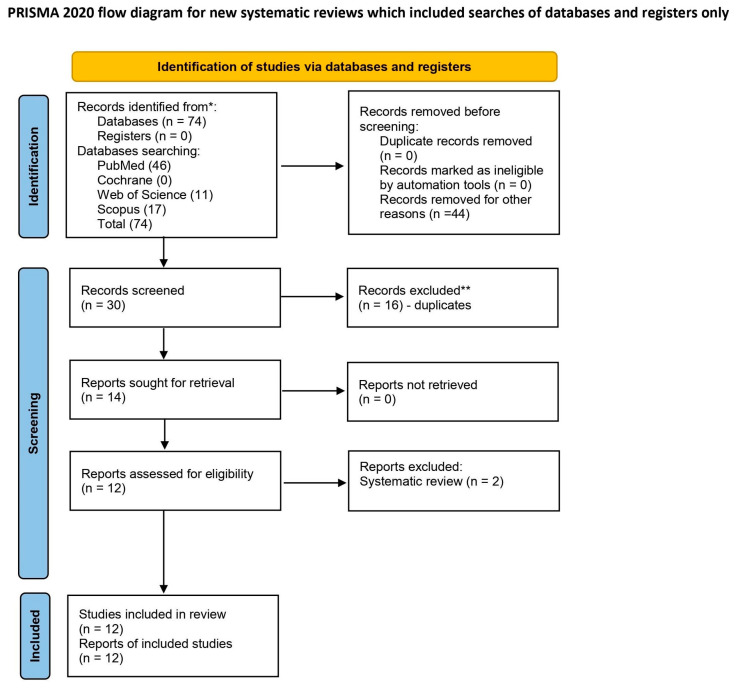
The PRISMA 2020 flow diagram [25].

The selection process for articles in the systematic review was carefully outlined following the PRISMA flow diagram (Figure 3) [25].

### 2.3. Eligibility Criteria

The review included studies that adhered to the following criteria: studies investigating the use of graphene materials in orthodontics, both in vitro and in vivo studies, studies published in English, and studies with a control group. The reviewers collectively decided to exclude studies that met the following criteria: non-English studies, opinion pieces, review articles and meta-analyses, letters to the editor, editorial papers, clinical reports, studies without full-text accessibility, and duplicated publications. No restrictions were imposed on the year of publication.

### 2.4. Information Sources, Search Strategy, and Study Selection

On 1 February 2023, electronic searches were conducted in the following databases: Pubmed, Cochrane, Web of Science, and Scopus. For the Scopus database, results were narrowed to titles, authors and keywords. Searches were limited to individuals and studies that met the eligibility criteria. The study was then supplemented with a literature search of the articles considered not found during the database search. Only articles with full-text versions available were considered.

### 2.5. Data Collection Process and Data Items

Two reviewers autonomously gathered data from articles that fulfilled the inclusion criteria. The extracted information was then entered into a standardized Excel file.

### 2.6. Assessing Risk of Bias in Individual Studies

At the initial stage of study selection, the titles and abstracts of each study were independently checked by the authors to minimise potential reviewer bias. The level of agreement among reviewers was determined using Cohen’s κ test [26]. Any differences of opinion on the inclusion or exclusion of a study were resolved through discussion between authors. 

### 2.7. Quality Assessment

Two independent reviewers (J.M., M.D.) evaluated the procedural quality of each study included in the article. The assessment criteria were based on the presence of key information related to the association of graphene oxide use in orthodontic treatment. To evaluate the study design, implementation, and analysis, the following criteria were used: a minimum group size of 10 subjects, the presence of a control group, a clear description of the performed procedure technique, the specific orthodontic adhesive used in the research, a biocompatibility test for graphene oxide, consideration of the type of orthodontic arch in the study, and analysis of the effect of graphene oxide on friction in the bracket–arch system, including parameters such as shear bond strength (SBS) and adhesive remnant index (ARI). The studies were scored on a scale of 0 to 9 points, with a higher score indicating higher study quality. The risk of bias was assessed as follows: 0–3 points denoted a high risk, 4–6 points denoted a moderate risk, and 7–9 points indicated a low risk. Any discrepancies in scoring were resolved through discussion until a consensus was reached.

## 3. Results

### 3.1. Study Selection

An initial search of the database identified 74 articles that were potentially eligible for the literature review. A first selection of article titles and abstracts allowed the exclusion of 44 articles as unrelated to the reviewed topic. Among the remaining 30 articles, there were 16 duplicates. Two non-research articles were rejected. Finally, 12 articles were qualified for the systematic review. All of the included studies were in vitro studies.

### 3.2. General Characteristics of the Included Studies

Twelve studies were included in this review. The general characteristics of each study (purpose of the study, control and study group, results, and conclusions) are presented in Table 1. 

Shear bond strength (SBS) and adhesive remnant index (ARI) coefficients are essential parameters for describing the mechanical properties of orthodontic adhesives [27]. In the research conducted by Maryam Pourhajibagher et al. [19], Roghayeh Ghorbanzadeh et al. [8], and Nozha M. Sawana et al. [6], no significant difference in ARI value was observed between the control samples and the modified experimental groups. However, in another study by Nozha M. Sawana et al. [28], orthodontic adhesive samples treated with 0.35 wt% Ag-GS (graphene sheets decorated with silver nanoparticles) showed the lowest ARI score among all the experimental groups after thermocycling. Additionally, in the study conducted by Mohammad Alnatheera et al. [16], the samples treated with 0.25 wt% SGO-modified adhesive (silanized graphene oxide) exhibited the lowest ARI scores.

According to the investigation by Roghayeh Ghorbanzadeh et al. [8] and Maryam Pourhajibagher et al. [19], Transbond XT with 5% wt. nGO (nanographene oxide) exhibited the highest shear bond strength (SBS) value. In the study conducted by Seung-Min Lee et al. [14], the SBS of the control group showed no significant difference compared to the BAG@GO (graphene oxide with a bioactive glass mixture) group, whereas adhesives with 1 wt% BAG@GO showed a small gain in SBS. In the Nozha M. Sawana et al. [28] study, there was no significant difference in SBS between 0.35 wt% Ag-GS and the commercial adhesive after 2 h of storage in distilled water. However, a notable difference in SBS was observed when the nanoparticle concentration was improved (0.55 wt% Ag-GS) compared to the commercial adhesive, indicating that higher nanoparticle concentrations reduced SBS. In the study conducted by Mohammad Alnatheera et al. [16], the samples treated with 0.25 wt% SGO-modified adhesive showed the highest mean SBS. Lastly, in the study by Nozha M. et al. [6], the effect of GNP-Ag (graphene nanoplatelets with silver nanoparticles) concentration on SBS was explored over a 24 h period of storage in distilled water. The findings revealed a gradual decline in SBS with an increase in GNP-Ag concentration in the experimental adhesive. This temporal insight highlights the importance of considering the long-term performance and stability of nanoparticle-infused adhesives in orthodontic treatment.

The research conducted by Maryam Pourhajibagher et al. [12,19] and Roghayeh Ghorbanzadeh et al. [8] demonstrated that the addition of nGO to Transbond XT led to a significant reduction in *S. mutans* colony counts, indicating antimicrobial properties. However, Maryam Pourhajibagher et al. [12] specifically noted that only the concentration of 10 wt% nGO showed a statistically significant decrease in the colony-forming units of test microorganisms after 60 days. The anti-microbial activity of the eluted components from the modified orthodontic adhesive discs against *S. mutans* was directly related to the concentration of nGO. Moreover, the study by Maryam Pourhajibagher et al. [19] revealed a gradual increase in biofilm inhibition with increasing nGO concentrations, without a concurrent increase in the failure rates of brackets. On the other hand, it was observed that concentrations higher than 5 wt% nGO significantly inhibited the growth of *S. mutans*, but this was accompanied by a decrease in the average bond strength of the adhesive to the enamel as the nGO concentration exceeded 5%. The ideal orthodontic adhesive (composite) should possess antimicrobial properties and be capable of inducing enamel remineralization without adversely affecting the bond strength of the brackets to the enamel.

The studies conducted by Maryam Pourhajibagher et al. [12,19] and Roghayeh Ghorbanzadeh et al. [8] revealed that adding nGO to Transbond XT resulted in a significant reduction in *S. mutans* colony counts, indicating its antimicrobial properties. Notably, Maryam Pourhajibagher et al. [12] specifically emphasized that only the concentration of 10 wt% nGO demonstrated a statistically significant decrease in the colony-forming units of test microorganisms after 60 days. The antimicrobial activity of the eluted components from the modified orthodontic adhesive discs against *S. mutans* was directly correlated with the concentration of nGO. Additionally, Maryam Pourhajibagher et al. [19] observed a gradual increase in biofilm inhibition with increasing nGO concentrations without a concurrent increase in the failure rates of brackets. However, it was also observed that concentrations higher than 5 wt% nGO led to significant inhibition of *S. mutans* growth, but this was accompanied by a decrease in the average bond strength of the adhesive to the enamel as the nGO concentration exceeded 5%. This highlights the importance of balancing antimicrobial properties with maintaining strong bonding to the enamel for an ideal orthodontic adhesive (composite), which should possess both antimicrobial capabilities and the ability to induce enamel remineralization without compromising the bond strength of the brackets to the enamel.

Nozha M. et al. [6] conducted a study with the objective of formulating and characterizing functionalized graphene nanoplatelets (GNPs) combined with silver nanoparticles (AgNPs) to evaluate the antimicrobial and mechanical properties of GNP-Ag-modified adhesives used in bonding orthodontic brackets. The research yielded noteworthy results regarding the viability of human gingival fibroblast (HGF) cells when exposed to the modified experimental adhesive (Transbond XT) [6]. The experimental adhesive containing 0.25 wt% and 0.5 wt% of GNP-Ag (graphene nanoplatelets with silver nanoparticles) showed low cytotoxicity, with cell survival rates exceeding 80%. However, after 48 h, the adhesive with 0.5 wt% of GNPAg exhibited considerable cytotoxic behaviour. On the other hand, the adhesive with 0.25 wt% of GNP-Ag demonstrated a substantial increase in antibacterial properties, making it suitable for bonding orthodontic brackets to the enamel surface without compromising bond strength.

In a separate study conducted by Nozha M. et al. [28], their primary objective was to modify orthodontic adhesive by skilfully incorporating graphene sheets adorned with silver nanoparticles (Ag-GS). After bonding the orthodontic brackets, they aimed to evaluate the ensuing mechanical and antibacterial properties. The investigation demonstrated a clear trend of decreasing relative microbiological viability with a proportional increase in the weight percentage (wt%) of nanoparticles in the adhesives. However, it was observed that the orthodontic adhesive containing 0.55 wt% of Ag-GS exhibited considerable cytotoxic behaviour after 48 h. These findings underscore the crucial significance of meticulously optimizing the nanoparticle concentration in orthodontic adhesives to ensure both biocompatibility and effective antimicrobial properties. In the study conducted by Mohammad Alnatheera et al. [16], their research focus was on modifying the Transbond XT (control adhesive) by skilfully incorporating 0.25 wt% and 0.5 wt% of SGO-modified adhesive (silanized graphene oxide). The 0.25 wt% SGO-modified adhesive group showed the most effective bactericidal properties and exhibited the least cytotoxicity when compared to the 0.5 wt% SGO-modified adhesive and Transbond XT. Furthermore, Jung-Hwan Lee et al. [29] investigated the antimicrobial-adhesive effects of PMMA with and without nGO incorporation. Specimens with higher amounts of nGO exhibited more robust anti-adhesion effects against all microbial species, and no significant cytotoxicity was observed when compared to the control group. While this innate nGO did not significantly enhance mechanical properties compared to the control, it did not exhibit any systemic toxic effects in humans from fully polymerized PMMA products, as reported in previous studies.

**Figure 4:**
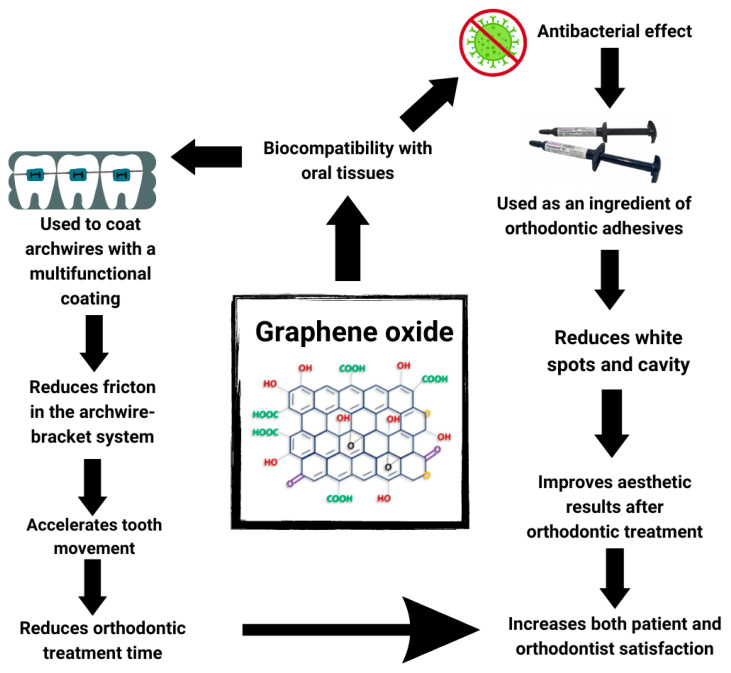
Figure illustrates the use of graphene oxide in orthodontic treatment (created with Canva.com (accessed on 1 September 2023)).

Pengfei Wang et al. [15] and Zonglin Pan et al. [30] conducted a comprehensive study exploring the fretting friction and wear behaviours of stainless steel archwires coated with a carbon film that contained embedded graphene sheets (GSEC). They specifically investigated how these GSEC-coated orthodontic stainless steel archwires performed in contact with untextured and microgroove textured stainless steel brackets within an artificial saliva environment. The research findings strongly suggested that the combined effect of the GSEC film on the archwire surface and the micro-groove textures on the brackets had a remarkable positive influence on the overall friction and wear characteristics of the stainless steel archwire–bracket sliding contacts. As a result, the study demonstrated great promise for utilizing GSEC-coated archwires in various clinical orthodontic treatment applications (Figure 4).

Zonglin Pan et al. [30] conducted a study with comparable outcomes. Their research demonstrated that GSEC film-coated archwires exhibited low friction coefficients and exceptional wear resistance when interacting with stainless steel brackets in artificial saliva environments. The authors concluded that applying the surface coating technique with GSEC film could lead to more effective and efficient orthodontic treatment. On the other hand, Danni Daia et al. [7] focused on graphene oxide (GO) coatings on NiTi (nickel titanium) alloys. Their study revealed that the coating did not sufficiently cover the substrate at low GO concentrations, providing only minor enhancements to NiTi alloy’s tribological and anti-corrosion properties. However, as the GO concentration increased, the GO coating exhibited significantly improved antibacterial activity. It was cautioned that higher GO concentrations might compromise the biocompatibility of GO-coated NiTi alloys. The authors suggested their study offered a controllable and straightforward process to enhance NiTi alloy surface properties, including corrosion resistance, friction resistance, and antimicrobial properties, while ensuring biocompatibility. Additionally, Delong Jiao et al. [6] conducted a series of experiments in vitro and in vivo (on mice), demonstrating that the application of GOG (gelatine reduced graphene oxide) can accelerate orthodontic tooth movement. The results indicated accelerated bone remodelling in the GOG-treated group, with enhanced osteoblasto-/osteoclasto-genesis and angiogenesis.

**Table 1:** General characteristics of the included studies.

Studies	Purpose of the Study	Control and Study Group	Results	Conclusions
Maryam Pourhajibagher et al. [19]	SBS and ARI scores of orthodontic adhesive were incorporated with nGO. The antimicrobial activities of the modified orthodontic adhesive were compared against *S. mutans*.	Transbond XT (3M Unitek, Monrovia, CA, USA) with 0 (as the control), 1, 2, 5, and 10 wt% nGO.	- The orthodontic adhesive containing 5 wt% nGO exhibited the highest concentration of nGO and shear bond strength (SBS) value. - It also showed no significant differences in adhesive remnant index compared to the control group. - SBS in the 1%, 2%, and 5% nGO groups was significantly higher than that in the 10% nGO group.	- Addition of 5 wt% nGO to the orthodontic adhesive can be deemed effective in reducing microbial count and biofilm formation without negatively impacting shear bond strength (SBS) and adhesive remnant index (ARI).
Delong Jiao et al. [18]	The aim of the study was to find out if gelatine reduced graphene oxide (GOG) accelerated orthodontic tooth movement.	In the experimental group, GOG solution was administered via buccal submucous local injection around the maxillary left first molar, while the control group received phosphate buffer saline (PBS) solution injection.	- The administration of GOG led to accelerated bone remodelling, promoting the generation of osteoblasts and osteoclasts as well as stimulating angiogenesis.	- Serial experiments in vitro and in vivo showed that application of GOG can accelerate orthodontic tooth movement.
Roghayeh Ghorbanzadeh et al. [8]	Physiomechanical and antimicrobial effectiveness of a novel orthodontic composite (OC-nGO) containing nGO following photodynamic therapy (PDT) and photothermal therapy (PTT) was compared against Streptococcus mutans.	Transbond XT (3M Unitek, Monrovia, CA, USA) with 0 (as the control), 1, 2, 5, and 10 wt% nGO.	- *S. mutans* metabolic activity and biofilm susceptibility were most affected by diode laser light when 5% wt. nGO was present for up to 150 days of rinsing. - Transbond XT with 5% wt. nGO exhibited the highest shear bond strength (SBS) value, along with potent antimicrobial and anti-biofilm activities. - All test concentrations of nGO in OC-nGO demonstrated adhesive remnant index (ARI) scores similar to the control group.	- Photo-activated 5% wt. OC-nGO can be used as an additive in orthodontic composites/adhesives to effectively control cariogenic bacterial biofilms.
Maryam Pourhajibagher et al. [12]	Antimicrobial and cytotoxic effects of a conventional orthodontic adhesive infused with varying concentrations of nanographene oxide (nGO).	Transbond XT (3M Unitek, Monrovia, CA, USA) with 0 (as the control), 1, 2, 5, and 10 wt% nGO.	- The modified orthodontic adhesive did not exhibit any cytotoxic effects on HGF cells. - The Transbond XT adhesive infused with 5% and 10% nGO significantly reduced the mean total viable counts of *S. mutans* for up to 30 days. - However, after 60 days, only the adhesive containing 10% nGO showed a statistically significant decrease in the colony-forming units of the test microorganisms.	- The antimicrobial activity against *S. mutans* in cariogenic biofilms is notably significant when using a modified orthodontic adhesive containing nGO at concentrations of 5% and 10%.
Seung-Min Lee et al. [14]	Mechanical and biological properties of orthodontic bonding adhesive enriched with graphene oxide and bioactive glass.	Transbond™ Supreme Low-Viscosity Light Cure Adhesive, 3M, Monrovia, CA, USA) with 0 (as the control), 1, 3, and 5 wt% of BAG@GO.	- There was no statistically significant difference in shear bond strength (SBS) between the control group and the BAG@GO group. - The adhesives with 1 wt% BAG@GO exhibited a slight increase in SBS. - However, the adhesive group with 5 wt% BAG@GO showed lower SBS, although this difference was not statistically significant.	- The mechanical properties of the orthodontic bonding adhesive enriched with graphene oxide and bioactive glass were suitable for clinical use. - The biological properties were found to be safe for application to patients.
Pengfei Wang et al. [15]	Fretting friction and wear behaviour of the graphene sheets embedded carbon (GSEC) stainless steel archwire in the archwire–bracket contact.	Graphene sheets embedded carbon (GSEC) films were produced with a high substrate bias voltage. The control group consisted of uncoated stainless steel archwire sliding against a conventional stainless steel bracket.	- The combined influence of the GSEC films on the archwires and the micro-groove textures on the brackets led to outstanding friction and wear performance in the archwire–bracket sliding contacts.	- The study suggested great potential of the GSEC films for clinical orthodontic treatment applications.
Jung-Hwan Lee et al. [29]	Antimicrobial-adhesive effects of PMMA with and without nGO (as the control) incorporation.	In nGO and nGO-incorporated PMMA (up to 2 wt%), the 3-point flexural strength and hardness were assessed. To examine the anti-adhesive effects, the experimental specimens were tested against four different microbial species. The control group involved bacteria only.	- Specimens containing higher levels of nGO exhibited more potent anti-adhesion effects against all microbial species. - None of the specimens showed any signs of cytotoxicity when compared to the control group, and there have been no reports of systemic toxic effects in humans from fully polymerized PMMA products. - However, the innate nGO did not significantly enhance the mechanical properties compared to the control.	- The data indicate that nGO-incorporated PMMA holds promise for various applications, including removable or provisional prosthodontics, as well as implantable biomaterials, thanks to its documented antimicrobial and anti-adhesive properties.
Nozha M. et al. [28]	Incorporating graphene sheets decorated with silver nanoparticles (Ag-GS) and investigating how these modifications impact the mechanical and antibacterial properties after bonding with orthodontic brackets.	Ag-GS was added to the orthodontic adhesive (Transbond XT orthodontic adhesive (3 M, Unitek, USA)) in two distinct concentrations (0.35 and 0.55 wt%). As a control adhesive, a Transbond XT adhesive was utilized.	- After 2 h of storage in distilled water, there was no significant difference in shear bond strength (SBS) between the 0.35 wt% Ag-GS modified adhesive and the commercial adhesive. - However, as the nanoparticle concentration increased (0.55 wt% Ag-GS), a noticeable decrease in SBS was observed compared to the commercial adhesive, indicating that higher nanoparticle concentrations negatively affected SBS. - The orthodontic adhesive with 0.55 wt% Ag-GS demonstrated significant cytotoxic behaviour after 48 h.	- Incorporating 0.35 wt% of nanoparticles significantly enhances the antibacterial capabilities of the orthodontic adhesives. - This concentration demonstrated promising outcomes for bonding orthodontic brackets to enamel without compromising the adhesive strength.
Mohammad Alnatheera et al. [16]	Creating and analysing silanized graphene oxide (SGO) nanoparticles and evaluating their spectral, microbiological, and mechanical properties when incorporated into orthodontic adhesive for bonding to orthodontic brackets.	Transbond XT (control adhesive) was modified by incorporating 0.25 wt% and 0.5 wt% SGO-modified adhesive.	- The samples treated with 0.25 wt% SGO-modified adhesive exhibited the highest mean SBS and the lowest ARI scores. - The 0.25 wt% SGO-modified adhesive group demonstrated the most potent bactericidal effect and the least cytotoxicity compared to the 0.5 wt% SGO-modified adhesive and Transbond XT groups.	- Incorporating 0.25 wt% of SGO into Transbond XT resulted in improved antimicrobial and mechanical properties, making it a promising option for bonding orthodontic brackets.
Zonglin Pan et al. [30]	Influence of coating stainless steel archwires with graphene sheets embedded carbon (GSEC) film on friction on archwire–bracket contact.	Carbon films were produced using an electron cyclotron resonance plasma sputtering system at substrate bias voltages ranging from +5 to +50 V. Uncoated stainless steel archwires were used as the control.	- When sliding against stainless steel brackets in artificial saliva environments, the GSEC film-coated archwires demonstrated low friction coefficients and high wear resistance.	- The application of the GSEC film surface coating technique is anticipated to enhance the effectiveness and efficiency of orthodontic treatments.
Nozha M. et al. [6]	Creating and analysing graphene nanoplatelets (GNPs) functionalized with silver nanoparticles (AgNPs) and assessing the antimicrobial and mechanical properties of the resulting GNP-Ag-modified adhesives when used to bond orthodontic brackets.	Graphene conjugated with Ag nanoparticles were incorporated into the Transbond XT orthodontic adhesive (3 M, Unitek, USA) at 0.25 wt% and 0.5 wt%. Unmodified Transbond XT was kept as a control group.	- The ARI values showed no significant difference between the control samples and the modified experimental groups. - After 24 h of storage in distilled water, the SBS decreased with an increase in GNP-Ag concentration in the experimental adhesive. - The study revealed a notable difference in the viability of HGF cells between the modified and unmodified experimental adhesive. - The experimental adhesive containing 0.25 and 0.5 wt% of GNP-Ag demonstrated low cytotoxicity, with the cell survival rate being above 80%. - However, after 48 h, the experimental adhesive with 0.5 wt% GNP-Ag exhibited significant cytotoxic behaviour.	- Incorporating silver nanoparticle-doped graphene nanoplatelets could serve as a potential antimicrobial modification for orthodontic adhesives. - Addition of 0.25 wt% of this composite resulted in a significant increase in antibacterial properties, making it a suitable choice for bonding orthodontic brackets to the enamel surface without compromising bond strength.
Danni Daia et al. [7]	Exploration of the impact of different concentrations of graphene oxide (GO) coatings for NiTi alloy on corrosion resistance, friction performance, and antibacterial properties.	Specially prepared samples were coated with 0 (as the control), 0.5, 2, or 5 mg/mL GO concentrations.	- At low GO concentrations, the coating on the substrate was insufficient, leading to limited improvements in the tribological and anti-corrosion properties of the NiTi alloy. - As the GO concentration increased, the antibacterial activity of the coating improved continuously. - However, higher GO concentrations may compromise the biocompatibility of the GO-coated NiTi.	- By applying a multifunctional GO nanocoating, the surface of NiTi can be enhanced to improve its corrosion resistance, friction resistance, and antimicrobial properties while ensuring biocompatibility.

SBS—shear bond strength; ARI—adhesive remnant index; GOG—gelatine reduced graphene oxide; HGF—human gingival fibroblast; BAG@GO—Graphene oxide (GO) with a bioactive glass (BAG) mixture; Ag-GS—graphene sheets decorated with silver nanoparticles; SGO—silanized graphene oxide; GNP-Ag—graphene nanoplatelets (GNPs) with silver nanoparticles (AgNPs).

### 3.3. Main Study Outcomes

Nine studies [6,7,8,9,12,14,16,18,20] demonstrated the antibacterial properties of graphene oxide, which can reduce the demineralisation of enamel during orthodontic treatment. Seven studies [6,8,12,14,16,18,20] showed that it is biocompatible with oral tissues. Three studies [4,7,15] showed that graphene oxide can reduce friction in the arch-bracket system. Two studies [16,20] showed that it can improve the mechanical properties of orthodontic adhesives by reducing ARI (Adhesive Remnant Index). Three studies [8,16,19] demonstrated that graphene oxide used in the appropriate concentration can also increase the SBS (shear bond strength) parameter. One research study [7] showed that it can increase the corrosion resistance. One research study [18] suggested that it can be used to accelerate orthodontic tooth movement. It can be used to coat the surface of orthodontic arches [4,6,15] and as an additive to orthodontic adhesives [6,8,12,14,16,19,20].

### 3.4. Quality Assessment

**Figure 5:**
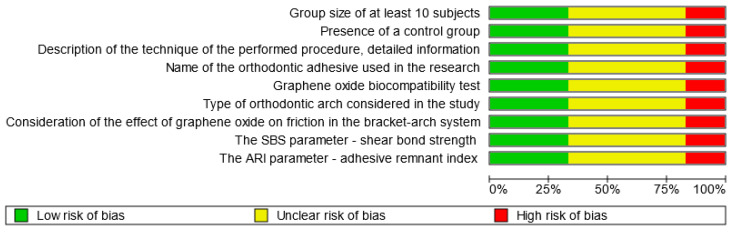
Distribution of bias risk of eligible studies (created with RevMan Web, Cochrane, UK).

Out of the articles included in the review, four [6,8,16,20] were deemed high-quality, with a score of 7/9 points. Two studies [9,18] were classified as low-quality. Additionally, six studies [4,7,12,14,15,19] were considered to have a moderate risk of bias, scoring between 4 and 6 points (Table 2). Distribution of bias risk of eligible studies is presented in Figure 5.

## 4. Discussion

In modern dentistry, tremendous efforts have been made to prevent oral diseases and promote oral hygiene [2]. Restorative materials with excellent biological properties, improved mechanical properties, and longer service life have always been sought [5]. Graphene oxide has been shown to possess antibacterial properties and biocompatibility, making it a safe and beneficial material for use in oral cavity biomaterials [21]. Several studies meeting the inclusion criteria in this review have demonstrated various potential applications of graphene oxide in orthodontic treatment. Some of these studies [6,8,19] found no significant difference in the adhesive remnant index (ARI) between the control and modified experimental groups. Conversely, other studies [16,20] reported that orthodontic adhesive samples modified with graphene oxide exhibited the lowest ARI scores among all the groups studied. Regarding shear bond strength (SBS), some authors [14,19,20] did not find a statistically significant difference between the study and control groups. However, other studies [5,8,16] suggested that Transbond XT modified with nGO showed the highest SBS value. In contrast, the study by Maryam Pourhajibagher et al. [19] indicated that SBS in the 10% nGO group was statistically lower than in the control group. Furthermore, the SBS value for the 1%, 2%, and 5% nGO groups was significantly higher than that in the 10% nGO group. Similarly, a significant reduction in SBS was observed with increasing nanoparticle concentration (0.55 wt% Ag-GS) [8]. Overall, graphene oxide has the potential to enhance the mechanical properties of orthodontic adhesives, provided that it is added at the appropriate concentration.

Numerous studies [6,7,8,9,12,14,16,18,20] have highlighted the antibacterial properties of graphene oxide, which can effectively reduce enamel demineralization during orthodontic treatment. Authors [14] have even suggested that the anti-demineralization effect increases as the concentration of BAG@GO (graphene oxide coated with bioactive glass) rises. Similar findings were reported by Maryam Pourhajibagher et al. [19], demonstrating a gradual increase in biofilm inhibition (decrease in *S. mutans* growth) with higher nGO concentrations, without compromising the bond strength of the brackets (SBS). On the other hand, studies conducted by Nozha M. et al. [6] and Nozha M. et al. [28] revealed significant cytotoxic behaviour for 0.5 wt% of GNPAg experimental adhesive and 0.55 wt% of Ag-GS orthodontic adhesive, respectively, after 48 h. These results underscore the importance of using graphene oxide in appropriate concentrations to avoid potential cytotoxicity and impairment of bonding strength in orthodontic adhesives.

Several studies [4,7,15] have demonstrated that graphene oxide (GO) can effectively reduce friction in the archwire–bracket system. For instance, GO can be used to coat stainless steel archwires with a graphene sheet embedded carbon (GSEC) film, leading to reduced friction in the archwire–bracket contact [4,15]. In another study by Danni Daia et al. [7], a multifunctional GO nanocoating was applied to the surface of NiTi to enhance its corrosion resistance, friction resistance, and antimicrobial properties. The authors suggested that the GO nanocoating could also improve the mechanical properties of orthodontic arches, such as corrosion resistance [7] and high wear resistance [6,15]. However, Danni Daia et al. [7] also pointed out that when the GO concentration was low, the GO coating inadequately covered the substrate, resulting in only slight improvements in the tribological and anti-corrosion properties of NiTi alloy. As the GO concentration increased, the GO coating exhibited enhanced antibacterial activity, but a higher concentration could compromise the biocompatibility of GO-coated NiTi. Therefore, the application of surface coating techniques using the GSEC film or GO nanocoating holds promise for making orthodontic treatment more effective and efficient. However, it is crucial to carefully control the concentration of graphene oxide coating to achieve a balance of desired surface properties. By optimizing the GO concentration, clinicians and patients can benefit from the improved performance of orthodontic materials.

Finally, it is worth noting that a study [18] presented compelling evidence for the potential of graphene oxide (GOG) to accelerate orthodontic tooth movement due to its unique properties. The authors demonstrated that the GOG-treated group exhibited accelerated bone remodelling with increased osteoblastic and osteoclastic activity, as well as enhanced angiogenesis [18]. The results consistently indicated the biocompatibility of GOG and its ability to stimulate bone marrow stromal cells (BMSCs) to promote bone remodelling and tooth movement [18]. 

These findings open up promising avenues for further research in this area. However, the limitations of the present systematic review need to be highlighted. Given the diverse array of articles, conducting a meta-analysis proved unfeasible. To enhance the assessment of this subject matter, further research is warranted, ideally with a larger sample size and encompassing both clinical and in vivo studies.

## 5. Conclusions

Thanks to its properties, graphene oxide may have prospects for use in orthodontic treatment. First of all, due to its biocompatibility, it can be used safely in the oral cavity. It has an antibacterial effect and can be added as an ingredient in orthodontic adhesives. It can reduce carious white spots and, consequently, cavities. Fewer white spots will also lead to improved aesthetic results after orthodontic treatment. Graphene oxide can be used to coat arches with a multifunctional coating. Reduction of friction in the archwire–bracket system improves the mechanical properties of orthodontic arches and increases corrosion resistance. Also, reduced friction contributes to faster tooth movement. Research also indicates graphene oxide’s ability to accelerate orthodontic tooth movement, which could speed up orthodontic treatment.

The development of technology gives better opportunities to both the patient and the orthodontist due to the new physicochemical, mechanical, and antibacterial properties of nanosized materials that can be used in coating orthodontic wires and producing orthodontic bonding materials. Not only can we control biofilm formation, reduce bacterial activity, and facilitate anticariogenic action, but also, through the desired tooth movement, shorten the treatment time. This offers great prospects for the further development of research using graphene oxide in orthodontic treatment, especially in vivo. 

## Figures and Tables

**Table 2:** Assessing risk of bias, presence (1) or its absence (0).

Criteria/Authors	Maryam Pourhajibagher [19]	Delong [18]	Roghayeh Ghorbanzadeh [8]	Maryam Pourhajibagher [12]	Seung-Min [14]	Pengfei Wang [15]	Jung-Hwan [29]	Nozha M. [28]	Alnatheera [16]	Zonglin [30]	Nozha M. [6]	Danni [7]
Group size of at least 10 subjects	1	0	1	1	0	0	0	1	1	0	1	0
Control group	1	1	1	1	1	1	1	1	1	1	1	1
Description of the technique of the performed procedure, detailed information, e.g., additional instruments supporting the procedure, duration of the procedure	1	1	1	1	1	1	1	1	1	1	1	1
Name of the orthodontic adhesive used in the research	1	0	1	1	1	0	0	1	1	0	1	0
Graphene oxide biocompatibility test	0	1	1	1	1	0	1	1	1	0	1	1
Type of orthodontic arch considered in the study	0	0	0	0	0	1	0	0	0	1	0	1
Consideration of the effect of graphene oxide on friction in the bracket–arch system	0	0	0	0	0	1	0	0	0	1	0	1
The SBS parameter—shear bond strength	1	0	1	0	1	0	0	1	1	0	1	0
The ARI parameter—adhesive remnant index	1	0	1	0	1	0	0	1	1	0	1	0
Total points	6	3	7	5	6	4	3	7	7	4	7	5
Risk of bias	Moderate	High	Low	Moderate	Moderate	Moderate	High	Low	Low	Moderate	Low	Moderate

## Data Availability

Availability of supporting data—the datasets used and/or analysed during the current study are available from the corresponding author on reasonable request.
